# The critical relationship between tacrolimus levels, acute kidney injury, and early chronic lung allograft dysfunction

**DOI:** 10.3389/frtra.2025.1704682

**Published:** 2026-01-07

**Authors:** Roman Hauber, Luca Kohlhepp, Ignaz Briegel, Tobias Veit, Jürgen Barton, Bruno Meiser, Christian Schneider, Teresa Kauke, Rudolf Hatz, Dominik J. Hoechter, Nikolaus Kneidinger, Jürgen Behr

**Affiliations:** 1 Department of Internal Medicine II, Neuwittelsbach Academic Hospital of the University Hospital LMU Munich, Munich, Germany; 2 Center for Artificial Intelligence and Data Science, JMU University of Würzburg, Würzburg, Germany; 3 Department of Medicine V, Comprehensive Pneumology Center Munich (CPC-M), German Center for Lung Research (DZL), University Hospital LMU Munich, Munich, Germany; 4 Transplant Center, University Hospital LMU Munich, Munich, Germany; 5 Division of Thoracic Surgery, University Hospital LMU Munich, Munich, Germany; 6 Department of Anaesthesiology, University Hospital LMU Munich, Munich, Germany; 7 Division of Pulmonology, Department of Internal Medicine, Lung Research Cluster, Medical University of Graz, Graz, Austria

**Keywords:** lung transplantation, chronic lung allograft dysfunction, clad, tacrolimus, kidney injury

## Abstract

**Background:**

Based on clinical observations, we hypothesized that tacrolimus (TAC) exposure and acute kidney injury (AKI) are associated with the development of chronic lung allograft dysfunction (CLAD) after lung transplantation (LTx).

**Methods:**

Of 827 lung transplant recipients treated between 2000 and 2018, 509 with complete data sets from the University Hospital of Munich (LMU) were included in this study. In the context of a 10% reduction in FEV_1_ (CLAD10), tacrolimus and renal function were examined descriptively, inferentially, and through confounder analysis with regard to the occurrence of CLAD10.

**Results:**

Of 509 LTx patients, 67 (13%) died within the first 2 years after LTx. Among these 67 patients, 38 (57%) developed CLAD10 within 2 years of LTx. In these patients, we observed a temporal pattern characterized by a peak in TAC levels, followed by AKI, and subsequently subtherapeutic TAC concentrations, which occurred a few weeks before the onset of CLAD10. The confounder analysis demonstrated a significant influence of renal failure and tacrolimus fluctuations on the hazards ratio for developing CLAD10.

**Conclusion:**

Our data suggest that a transient decline in TAC serum concentrations, often caused by a TAC-induced AKI, may trigger the onset of CLAD10 and subsequently elevate the risk of premature death.

## Introduction

Despite advances in lung transplantation (LTx), long-term survival rates remain low compared to other solid organ transplants, and chronic lung allograft dysfunction (CLAD) is the leading cause of mortality (1–4).

Despite improvements in perioperative management, up to 20% of patients die within the first year after LTx, and approximately 20% develop CLAD within 2 years (4, 5). To achieve the best possible outcomes, it is essential to better understand the mechanisms and potential complications of the procedure (1–4, 6).

While CLAD is generally described as a multifactorial event, the variables contributing to its development remain insufficiently understood (3–8). Unlike other transplanted organs, the lung is constantly exposed to environmental air containing microbes, noxious particles, and gases, rendering it particularly vulnerable to external influences, such as infections and air pollution. Following LTx, the immune system must be downregulated appropriately to prevent acute rejection and CLAD while still allowing for an appropriate immunologic defense against microbial pathogens. Immunologic and non-immunologic risk factors for CLAD have been identified with variable levels of evidence (9–11). The most important immunologic risk factors include acute rejection, lymphocytic bronchiolitis, and medication non-compliance, all of which can lead to insufficiently low TAC levels (10, 12–15). Available evidence supports the concept that maintaining optimal and stable immunosuppression is a key determinant of long-term survival following LTx (10).

Lung function and renal function are interlinked in several ways in LTX patients. This relationship involves blood pressure regulation, fluid homeostasis, acid–base balance, and drug clearance (7, 16). Moreover, both organs are interconnected through major immune system-mediated processes and inflammatory reactions (7, 16–18). Lertjitbanjong et al. reported that the incidence of acute kidney injury (AKI) in lung transplant patients exceeds 50%, demonstrating its significant association with patient survival (16). AKI and renal failure are common after lung transplantation, with 9.3% of affected individuals requiring renal replacement therapy (16). Impaired kidney function is also associated with decreased survival after LTx (16).

Calcineurin antagonists (CNI) like TAC or cyclosporin-A (CSA) are key immunosuppressive agents used after LTx but have profound nephrotoxic effects, often causing AKI and chronic kidney disease in transplant recipients (8, 19, 20). Notably, TAC and CSA exert vasoconstrictive effects on both the afferent and efferent vessels of the kidney, eventually leading to reduced microperfusion and a diminished glomerular filtration rate (GFR) (21, 22). Progressive renal impairment leads to a vicious cycle of decreasing renal function and increasing nephrotoxic CNI concentrations, which exacerbates interstitial kidney injury and eventually leads to renal failure (21, 22). The causes of increased CNI concentrations are complex and may include dosing errors, drug–drug interactions, exsiccation, and patient non-adherence (22).

Both CLAD and AKI are associated with increased mortality. While the detrimental impact of low TAC levels and high interpatient variability in TAC plasma concentrations on CLAD development is well-documented, the association between kidney function, TAC levels, and incidence of CLAD has not been investigated (23–25). Based on clinical observations, we hypothesized that an underlying relationship exists between TAC levels, kidney function, and the onset of functional decline of lung allografts.

## Methods

### Data and design

In this retrospective cohort study, routine data on GFR, TAC levels, and lung function from LTx patients in the LMU lung transplant program between 2000 and 2018 were analyzed. The data were extracted from the LMU data repository on 13 March 2021. We considered all data available between 1 January 2000 and 13 March 2021, except for re-lung, heart–lung, and liver–lung transplantations. Data prior to 2000 were too sparse for inclusion. The study was approved by the local ethics committee of LMU Munich (project number 23-1003).

We provide data on the occurrence of CLAD for the entire period. Post-transplant care schedules include closer follow-up during the first 2 years, resulting in a higher density of available data per patient. In addition, patients tend to acquire fewer new comorbidities during the first 2 years after transplantation. Consequently, our analysis is confined to a 2-year period following LTx to enhance the robustness of our findings against potential confounding factors. To gain a more protacted perspective, survival data for a period of 5 years were analyzed using a Cox regression model.

Administration of TAC, mycophenolate mofetil (MMF), and prednisolone was standard practice for maintaining immunosuppression in lung transplant recipients at the study center throughout the observation period. MMF dosing and blood levels were not included in this analysis. Three laboratory blood markers were defined as indicators of renal injury (RI):

absolute creatinine serum concentration >1.2 mg/dL;
estimated GFR<60 mL/min, calculated using CKD-EPI (2021); and
creatinine difference >0.3 mg/dL within 48 h.

For the assessment of AKI, we excluded the first 7 days after transplantation, as perioperative AKI often occurs with remission and is mainly related to surgical factors.

Because pre-existing renal impairment may contribute to subsequent declines in kidney function, renal function values from up to 2 weeks prior to LTx were also included in the analysis, in accordance with the predefined definition.

Differences between single-lung (SLTx) and double-lung transplantation (DLTx) recipients were specifically assessed, as these groups may differ in baseline characteristics, comorbidities, perioperative morbidity and mortality, and relevant laboratory parameters.

CLAD20 was defined according to current consensus criteria and was designated when the “definite” definition was met, meaning that the decline persisted for at least 3 months (20). Arjuna et al. noted that even a persistent FEV_1_ reduction of ≥10% (CLAD10) represents a relevant change in FEV1 that exceeds the daily variability (10). Due to the substantially larger data set, we evaluated the data on the occurrence of CLAD10. Regarding the chronology of events, CLAD10 was considered to begin on the day when an FEV_1_ decline of >10% was first observed and persisted for >3 months, analogous to CLAD20 (5, 26).

Survival data for the first 2 years (730 days) after transplantation were available for all patients. For patients who died, the date of death after LTx was recorded; for patients requiring retransplantation, the date of retransplantation served as the survival date. The standard procedure at the contributing center requires that tacrolimus dosage be regularly adjusted based on laboratory results obtained from blood samples submitted by patients. The standard procedure involves no induction therapy, with a target range for tacrolimus of 12–15 ng/mL during the first year after transplantation. This range decreases to 10–12 ng/mL in the second year and continues to decline annually thereafter. Individual target ranges are tailored to the specific needs of each patient, and high rates of in-range measurements are consistently achieved. To control for changes in the target corridor and enhance robustness against fluctuations in target ranges according to the standard procedure, deltas between previous measurements are calculated.

### Statistics

We used standard descriptive statistics and Kaplan–Meier estimates to provide an overview of data dynamics. Statistical analysis and rendering were performed using R and Python. Due to the exploratory nature of our study, we kept statistical testing to a minimum.

We conducted an exploratory analysis with graphical visualization to consider the time-adjusted sequence of events surrounding the onset of CLAD10. Therefore, we standardized the data to the occurrence of CLAD10 (CLAD10 as *t* = 0) and chose a window of 60 days before and after CLAD10. TAC levels, serum creatinine concentrations (CREA), and calculated GFRs were analyzed in the timely context of CLAD10. *Y*-values represent TAC, GFR, and CREA after subtracting the baseline at *t* = 0; for example, a positive TAC-value at *t* = −30 implies a relatively higher serum level at that time point. To reduce noise and control for sparse data, each data point was binned at 2-day intervals, and the value calculated at *t* = 0 was subtracted from all measurements. Consequently, the *y*-axis is labeled “Δ”.

We conducted a subgroup analysis of patients who died within 2 years after LTx and analyzed the relationship between TAC and CREA/GFR. More specifically, we analyzed the trajectories of TAC and CREA/GFR levels in the period leading to CLAD10 using Mann–Whitney *U*-tests. Time intervals of 12 and 30 days were used for the statistical calculations to ensure sufficient data points for robust comparisons. The comparison areas were fitted to provide a statistical basis for the visual impression of differing measurement levels.

To account for potential confounding variables, we conducted a confounder analysis using multivariable regression models. Effect estimates (hazards ratios) and 95% confidence intervals were calculated and graphically summarized in a forest plot. We analyzed renal impairment, as well as patients with relevant outliers of TAC levels prior to CLAD-defined as having more than 25% of measurements outside the target therapeutic range established at our center. In addition, we included the type of LTx and available findings from transbronchial biopsy, cytomegalovirus (CMV) viremia, and CMV risk at the time of transplantation.

## Results

A total of 14,225 FEV_1_ values were included in the long-term analysis of CLAD10. In addition, 54,696 renal function measurements and 84,183 TAC level measurements were available. TAC measurements significantly outnumbered creatinine measurements because TAC monitoring required routine submission of blood samples to the transplant center, whereas CREA was not mandatory.

### Excluded patients

A total of 318 patients were excluded from this study because they had an insufficient number (<3) of pulmonary function tests (PFTs), which prevented the analysis of CLAD10 or CLAD20. For transparency, a comparison of the population with a complete data set to the 318 excluded patients is presented in Table 1. Notably, 50.3% of the excluded patients succumbed to their condition within 2 years, with a median time to death of 128 days. Over the subsequent 5 years, an additional 33 patients with limited PFT data died, resulting in a total of 193 deaths among patients with missing or limited PFT data within 5 years. In summary, the excluded group exhibited a significantly younger demographic, a higher proportion of male patients, and a shorter survival time compared with those in the full data set.

**Table 1:** Comparison of the population with complete data set available (*N* = 509, left column) and patients excluded (*N* = 318) due to incomplete data.

Variable	PFT 3 or more	PFT less than 3	*p*-Value^b^
	*N* = 509^a^	*N* = 318^a^	
Survival (days)	781 (506, 1,237)	166 (38, 573)	<0.001
2-year survival	442 (86,8%)	160 (50,3%)	
5-year survival	353 (69.4%)	193 (60.7%)	
Age at LTx (years)	54 (45, 60)	52 (41, 59)	0.043
Sex			<0.001
Female	269 (53%)	130 (41%)	
Male	240 (47%)	188 (59%)	
Diagnosis			0.3
CF/bronchiectasis	70 (14%)	57 (18%)	
COPD/emphysema	151 (30%)	86 (27%)	
Fibrosis	136 (27%)	87 (27%)	
Miscellaneous	130 (26%)	80 (25%)	
PAH group 1 (Nice)	22 (4.3%)	8 (2.5%)	
Type of LTx			0.11
dLTx	330 (65%)	218 (69%)	
sLTx left	87 (17%)	57 (18%)	
sLTx right	91 (18%)	39 (12%)	
Creatinine at LTx (mg/dL)	0.80 (0.70, 1.00)	0.85 (0.70, 1.00)	0.5
GFR at LTx (mL/min)	95 (80, 106)	98 (81, 110)	0.13

Statistical analysis was performed using the Wilcoxon rank-sum test or Pearson's chi-square test.

a Median (Q1, Q3); *n* (%).

b Wilcoxon rank-sum test; Pearson's chi-square test.

### Lung function and CLAD

**Figure 1:**
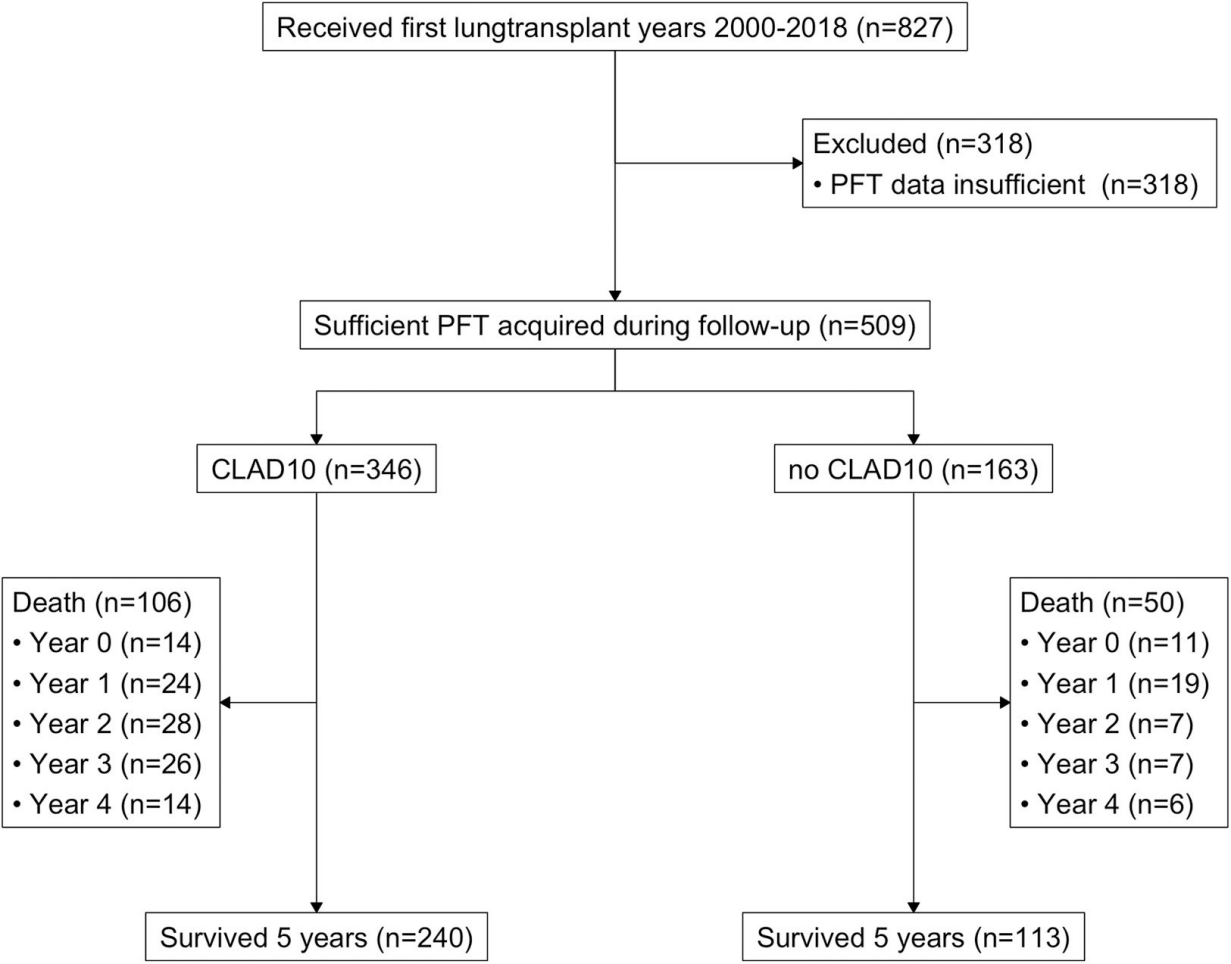
CONSORT diagram showing patient disposition

**Figure 2:**
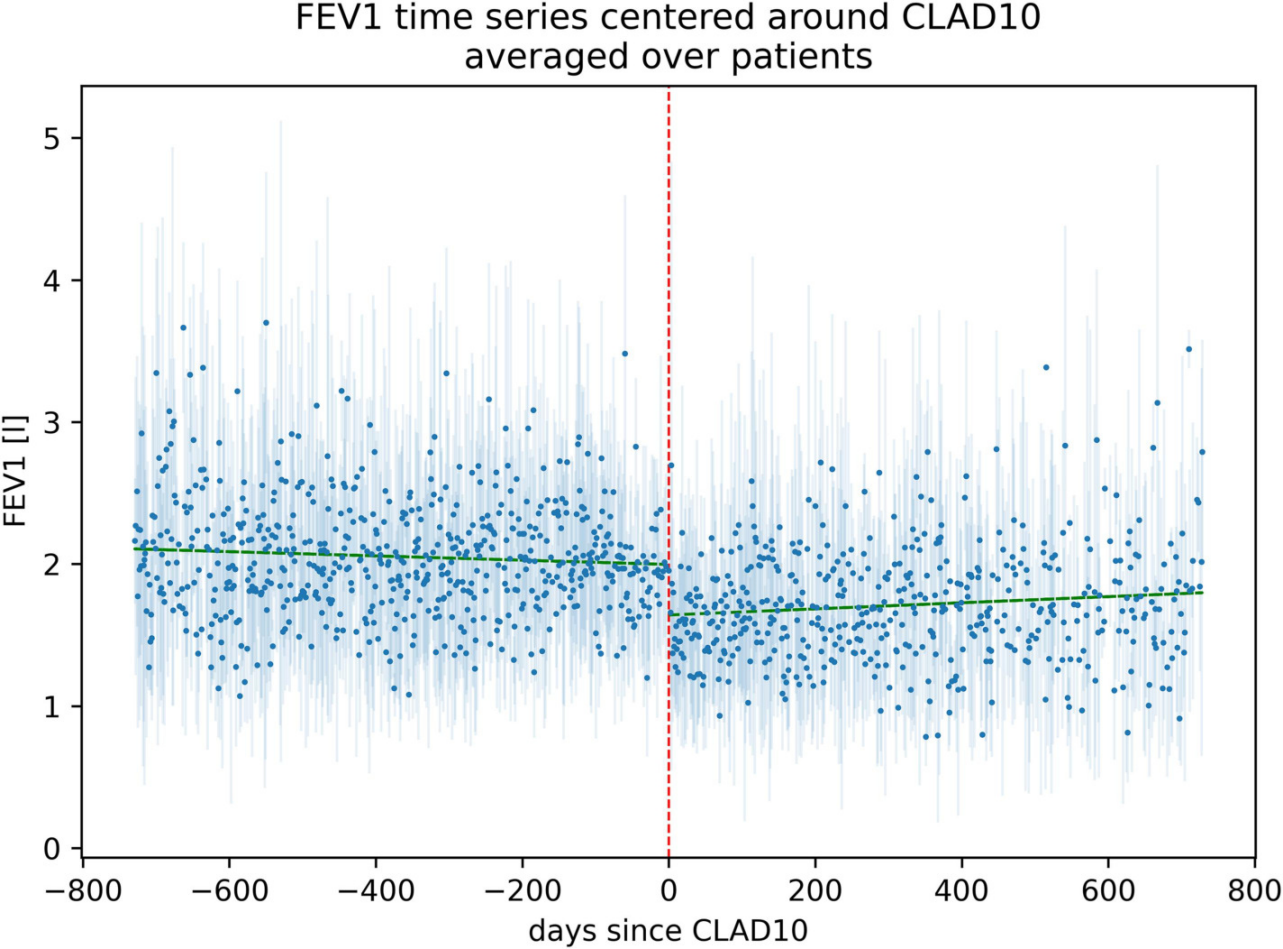
FEV_1_ measurements before and after the onset of CLAD10. The figure shows the averaged daily FEV_1_ values for all patients who developed CLAD10, mapped in relation to the time of first CLAD10 diagnosis, covering the period from 2 years (730 days) before to 2 years after CLAD10 diagnosis (light blue). The dashed red line marks the time of CLAD diagnosis. The green line shows the marginal mean decrease in FEV_1_ before CLAD and the marginal mean increase in FEV_1_ after CLAD10.

Of 827 primary lung transplant recipients, 509 patients had sufficient FEV_1_ data for analysis of CLAD10 (complete data set; see Figure 1). All of these patients also had available data on renal function and survival. Figure 2 presents an overview of the evolution of FEV_1_ during the 2 years preceding and the 2 years following the onset of CLAD10.

During the first 5 years after LTx, a total of 346 patients developed CLAD10. Of these, 293 patients were initially diagnosed with CLAD10. In addition, 53 patients were primarily detected with CLAD20, suggesting that a prior diagnosis of CLAD10 may have been missed. Furthermore, 182 patients with primary CLAD10 subsequently developed CLAD20 (62% of 293). Thus, a total of 235 patients developed CLAD20.

**Figure 3:**
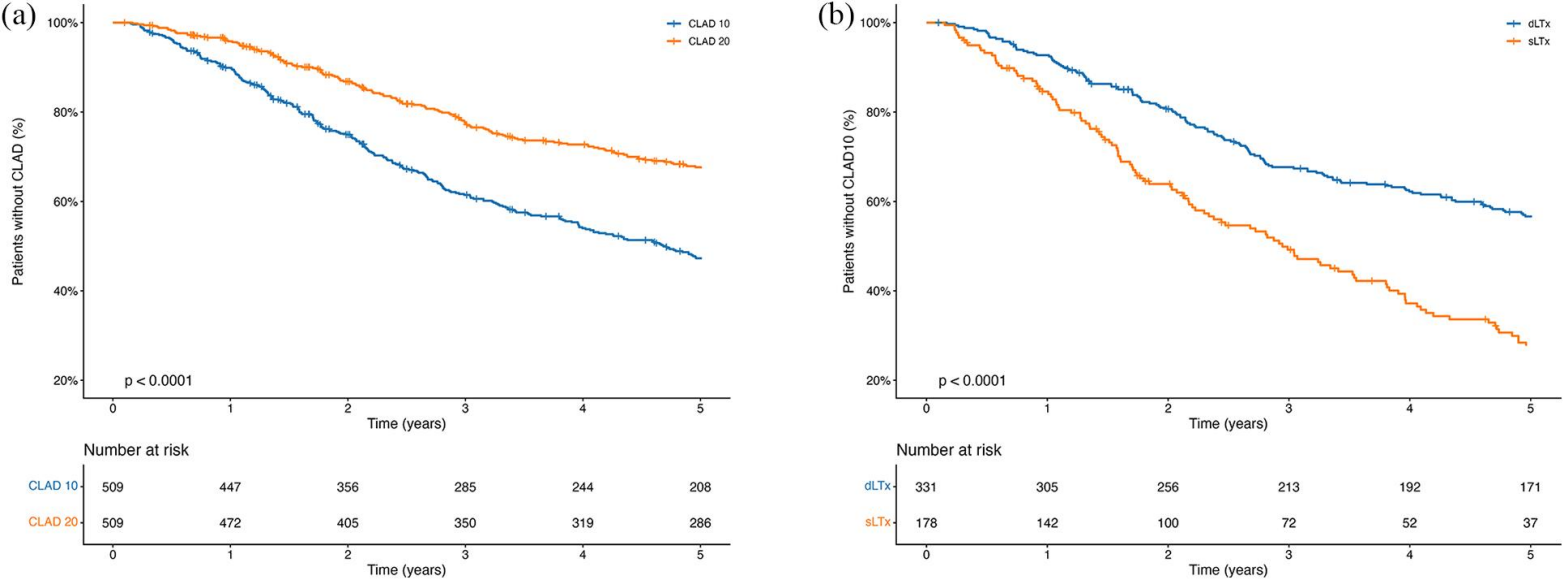
Occurrence of CLAD10 and CLAD20 up to 5 years after LTx. **(a)** Kaplan–Meier curves showing the proportion of patients without CLAD10 stratified by DLTx and SLTx over a 5-year follow-up, including all patients with complete data available (*n* = 509). Risk tables are provided. **(b)** Kaplan–Meier curves showing the proportion of patients without CLAD10 or CLAD20 over a 5-year follow-up, including all patients with complete data available (*n* = 509). Risk tables are provided.

The median time to diagnosis was 2.7 years for CLAD10 and 3.3 years for CLAD20. CLAD10 and CLAD20 were first detected 55 days and 61 days after LTx, respectively. Table 2 provides an overview of patient characteristics across four relevant subgroups. Figure 3a presents an overview of CLAD10 and CLAD20 occurrence during the first 5 years following LTx, and Figure 3b compares the development of CLAD10 between DLTx and SLTx patients. Both figures include associated risk tables.

**Table 2:** Summary of patient characteristics by age, sex, cause of lung failure, type of transplant, time to CLAD10/20, and renal function.

Character	Total	Complete data set	dLTx	sLTx	*p*-Value
	*N* = 827	*N* = 509	*N* = 331	*N* = 178	
Age at LTx (years)	53 (44–60)	54 (45–60)	48 (39, 56)	60 (56, 63)	<0.001
Sex					0.3
Female	399 (48%)	269 (53%)	180 (54%)	89 (50%)	
Male	428 (52%)	240 (47%)	151 (46%)	89 (50%)	
Diagnosis					<0.001
Fibrosis	223 (27%)	136 (27%)	81 (24%)	55 (31%)	
COPD/emphysema	237 (29%)	151 (30%)	64 (19%)	87 (49%)	
CF/bronchiectasis	127 (15%)	70 (14%)	68 (21%)	2 (1.1%)	
PAH group 1 (nice)	30 (4%)	22 (4.3%)	22 (6.6%)	0 (0%)	
Miscellaneous	210 (25%)	130 (26%)	130 (26%)	96 (29%)	
Time to CLAD10 (years)	2.7 (1.4–5.2)	2.7 (1.4, 5.3)	3.0 (1.7, 6.2)	2.2 (1.2, 4.1)	<0.001
Time to CLAD20 (years)	3.2 (1.9–6.5)	3.3 (1.9, 6.9)	3.7 (2.1, 7.8)	3.2 (1.5, 6.0)	0.039
Renal impairment pre-Tx	88 (11%)	49 (9.6%)	38 (12%)	11 (6,2%)	0.17
Creatinine at LTx (mg/dL)	0.9 (0.7–1)	0.85 (0.70, 1.00)	0.85 (0.73, 1.03)	0.85 (0.70, 0.95)	0.14
Creatinine at last observation (mg/dL)	1.5 (1.1–2.1)	1.60 (1.10, 2.40)	1.45 (1.10, 2.25)	1.70 (1.35, 2.70)	0.013
GFR at LTx (mL/min)	94 (78–105)	93 (80, 106)	95 (80, 109)	91 (79, 101)	0.023
GFR at last observation (mL/min)	50 (31–73)	45 (27, 68)	49 (29, 73)	39 (21, 56)	0.006

Data are presented as *n* (% of *N*) or median (IQR).

### Renal impairment

In the 14 days preceding LTx, 9.6% of patients demonstrated impaired renal function. Seven days after LTx, the median (interquartile range) serum creatinine was 0.9 mg/dL (0.7–1.1) and the median glomerular filtration rate was 93.8 mL/min (63.8–107). Approximately 56% of patients developed renal impairment within the first month after LTx. During the first 2 years of follow-up, renal impairment was observed in 96.5% of the population, in at least one of the recorded measurements. The median (interquartile range) time to the first occurrence of renal impairment was 227 days (121.5–339.5).

### Survival data

Survival rates were analyzed for patients with sufficient PFT data. Among these patients, 86.8% survived for up to 2 years and 69.4% of patients survived for up to 5 years following LTx. The median time to death was 440 days in the 2-year survival group and 781 days in the 5-year survival group. For transparency, we include the survival rates of the total cohort of 827 patients, which were 73.2% at 2 years and 57.8% at 5 years.

### DLTx vs. SLTx

SLTx patients were significantly older than DLTx patients (60 vs. 48 years). Their diagnosis distribution also differed significantly from the DLTx group: 49% of SLTx patients had COPD/emphysema, while only 2% had CF/bronchiectasis. In addition, SLTx patients exhibited a significantly earlier onset of CLAD10, as presented in Table 2.

### Relationship between tacrolimus, renal function, and CLAD10

This analysis focused on CLAD10 due to its higher event rate and its predictive role for manifest CLAD. Since data density is higher in the first 2 years than in subsequent years, and since effects may be obscured by confounding variables over a long period of time, we restricted the analysis to data from the first 2 years after LTx.

**Figure 4:**
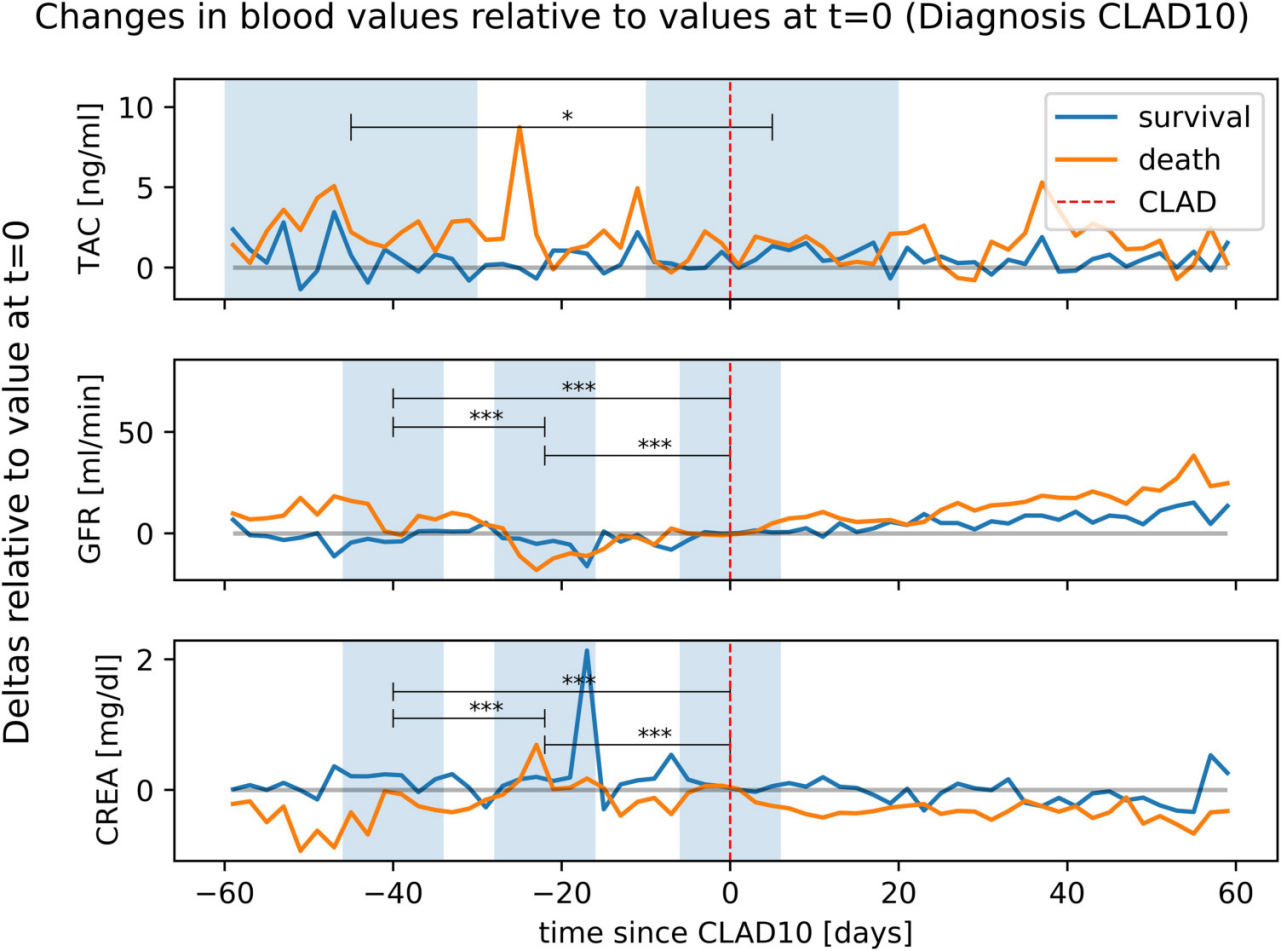
Changes in TAC, GFR, and CREA over a 60-day period surrounding the first diagnosis of CLAD10 (i.e., *t* = 0). The graph visualizes the TAC/CREA/GFR values in the period leading to CLAD. The *y*-values represent the magnitude by which the level deviates from the reference value at *t* = 0. Patients who survived or died were analyzed separately. Mean serum values for TAC [TAC (survival) = 8.2 ng/mL, TAC (death) = 10.1 ng/mL], and CREA [CREA (survival) = 1.9 mg/dL, CREA (death) = 2.0 mg/dL] at *t* = 0 (t0, i.e., onset of CLAD10) were subtracted from all respective measurements before and after t0. The *y*-values represent the deltas by which the level positively or negatively deviates from the reference value and not the absolute concentrations. Upper section (TAC): Two comparison windows in light blue (each 30 days, before and after AKI) illustrate changes in TAC levels. Middle and lower sections (CREA/GFR): 12-day comparison windows in light blue visualizes stable levels, decline, and recovery (before, during, and after AKI). Statistical comparisons were performed only within the group of patients who died. *p < 0.05, **p < 0.01, ***p < 0.001.

Among patients who developed CLAD10 and died within the first 2 years, a specific pattern emerged. The mean TAC level calculated over a 30-day period set around the day of CLAD10 diagnosis was significantly lower than that recorded during the 30-day period 4 weeks prior to the onset of CLAD10 (12.4 ± 5.9 ng/mL vs. 11.2 ± 5.9 ng/mL, *p* < 0.05) (Figure 4).

As shown in Figure 4, patients with CLAD10 who did not survive the first 2 years after LTx had higher TAC levels in the window preceding CLAD10 onset than patients with CLAD10 who survived (12.4 ± 5.9 ng/mL vs. 9.9 ± 5.3 ng/mL, *p* < 0.01). Among the patients with high TAC levels who did not survive the first 2 years after LTx, a decrease in TAC levels was followed within several days by a significant reduction in GFR (54.7 ± 22.5 mL/min to 38.1 ± 22.5 mL/min, *p* < 0.01). Approximately 3 weeks later, the FEV_1_ showed a sustained decrease of >10%, indicating the onset of CLAD10.

### Confounder analysis

**Figure 5:**
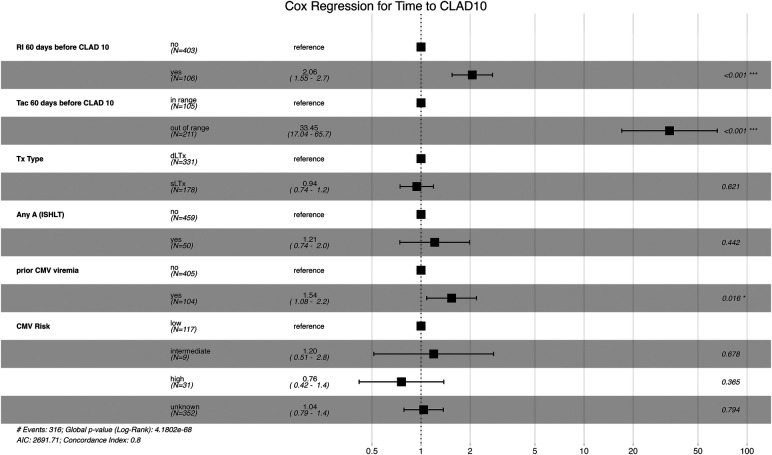
Forest plot of adjusted hazards ratios for potential confounders. The plot displays hazards ratios from the multivariable Cox regression model incorporating the following variables: markers of renal impairment, unstable tacrolimus levels prior to the onset of CLAD10, CMV risk, history of CMV viremia, history of ISHLT grade A1 or above in transbronchial biopsy, and treatment type (SLTx vs. DLTx). Each line represents the adjusted hazards ratio, along with its 95% confidence interval and *p*-value for the respective variable included in the confounder analysis.

As illustrated in Figure 5, we applied a multivariate Cox regression model and calculated hazards ratios to evaluate the risks associated with renal impairment, tacrolimus levels, and a history of acute rejection or CMV viraemia. This analysis allows for a visual comparison of the adjusted effects. The model included markers of renal impairment, unstable tacrolimus levels prior to the onset of CLAD10, CMV risk, history of CMV viremia, history of ISHLT grade A1 or above on transbronchial biopsy, and treatment type (SLTx vs. DLTx). CMV viremia, defined as viral loads exceeding 1,000 copies/mL, demonstrated a higher risk of developing CLAD10 (*p* < 0.016). However, the strongest effects observed were the presence of renal impairment and measured outliers in tacrolimus levels, both of which were highly significant (*p* < 0.001) in terms of their influence on the timing of CLAD10. Renal impairment was associated with a hazards ratio of 2.06, while derailed tacrolimus levels were associated with a hazards ratio of 33.45.

## Discussion

Our data suggest that a brief, transient decline in TAC levels below the therapeutic threshold is associated with a significant risk of subsequently developing CLAD10. In a subgroup of 38 patients who developed CLAD10 and died within 2 years after LTx, we observed a specific sequence of events, in which elevated TAC levels were followed by AKI, which was accompanied by an abrupt decline in TAC plasma concentrations, and CLAD10 emerged within approximately 20 days. We hypothesize that the development of AKI triggered the abrupt cessation or reduction of TAC dosing, leading to a decrease in TAC plasma concentration.

The 2- and 5-year survival rates of our cohort, at 73.2% and 57.8%, respectively, are in line with those reported in the literature from other European countries (27). Regarding the occurrence of renal impairment, our results are consistent with the literature, indicating that about 60% of transplanted patients suffer renal impairment in the first month post-transplant and almost all patients within the first 2 years after LTx (16). The time to manifest CLAD (i.e., CLAD20) in our cohort (3.3 years) is also comparable to that reported in previously published studies for other cohorts (26, 28). Altogether, our findings confirm that our cohort is representative of a contemporary LTx population.

Most patients who could not be evaluated due to a lack of PFT data died within the first 2 years (160 out of a total of 193 in 5 years). In this subgroup, incomplete data were attributed to early death, disability, or primary graft dysfunction.

While a persistent ≥20% decline in FEV_1_ is generally accepted as the definition of manifest CLAD, ample evidence suggests that this classification may be too late to identify the early stages of manifest CLAD (10, 29). A persistent ≥10% decline in FEV_1_ is already indicative of manifest CLAD and is associated with increased mortality (10, 29). In our study, 62% of patients who developed CLAD10 subsequently progressed to CLAD20, suggesting that CLAD10 could allow for earlier detection of graft failure and foster the development of timely interventional strategies.

Our findings for SLTx and DLTx recipients are consistent with common practice and the literature: older patients frequently present with more comorbidities, and single-lung transplantation is less invasive and may allow for better allocation of scarce donor organs to patients with limited life expectancy. In this context, the higher prevalence of COPD in the SLTx group is intuitive. Although mean survival after SLTx did not differ significantly in our cohort, a trend toward poorer outcomes may be discerned. Despite their older age and greater comorbidity burden, SLTx patients did not exhibit a higher prevalence of pre-existing renal failure prior to transplantation compared with DLTx recipients.

In patients with CLAD10 who survived less than 2 years after LTx, increased TAC levels seemed to induce AKI weeks before the onset of CLAD10. Comparatively, lower TAC levels after renal injury appeared to coincide with the onset of CLAD10.

The main management approach for AKI related to CNI toxicity includes lowering CNI serum concentrations, either by dose reduction or by pausing CNI administration. Our analysis indicates short-term iatrogenic dose reductions in CNI ahead of CLAD10. As a result, the CNI serum concentration may decline significantly below the desired target, thereby inducing a transient gap in patient immunosuppression. Interestingly, in a monocentric large LTx cohort, the severity of postoperative AKI was identified as a strong independent predictor of subsequent chronic kidney disease and was linked to long-term mortality (30). Whether such a short gap in immunosuppression may trigger acute rejection or contribute to the development of CLAD10 has not yet been systematically investigated.

Our study suggests that renal failure and derailed TAC levels are associated with the manifestation of CLAD10, leading to the following hypothesis: In select patients, renal failure can result in a cumulatively elevated concentration of tacrolimus, which may subsequently lead to further renal injury and hospitalization. Overcompensation of this effect by reducing tacrolimus dosage may contribute to an earlier onset of CLAD.

At the same time, it is possible that these observations are partly subject to a type of selection bias, as patients with renal failure or derailed TAC levels are often monitored more closely and may receive more intensive follow-up care. However, elevated TAC levels can have various adverse effects, most prominently nephrotoxicity, which can be aggravated by any pre-existing renal impairment and may potentially lead to a vicious cycle of chronic organ fibrosis. In addition, there is an increased susceptibility to infections and malignancies.

There are multiple possible explanations for the sudden increase in TAC levels in these patients, including accidental overdosage, exsiccation, or drug–drug interactions. Interestingly, GFR and creatinine recover after the onset of CLAD10, despite no corresponding change in TAC levels. This may also be due to improved volume management and patient guidance, which suggests the need for enhanced patient education and an emphasis on the multifactorial nature of CLAD10 pathogenesis. Our findings of a relevant interaction between TAC, AKI, and the development of CLAD10 are supported by regression analyses presented in the Supplementary Material. Our observation aligns with other reports linking TAC level fluctuations to the onset of CLAD10, which is likely driven by insufficient immunosuppression (12).

The confounder analysis showed a tendency, although not significant, for an earlier onset of CLAD10 in patients at high risk for CMV. Due to the multiplicity of risk factors, the retrospective nature of this study, and the limited data on the treatment of CMV viremia, we were not able to perform an in-depth analysis of the contribution of CMV viremia and antiviral therapy to the development of CLAD10 and CLD20.

### Limitations

Our study has limitations due to its monocentric and retrospective nature, which inherently carries a risk of bias. The data were extracted from routine clinical records. Laboratory data were collected according to the individual patient needs, are therefore sampled unevenly, and do not follow a standardized study protocol. Nonetheless, the statistical and descriptive correlations observed in this large data set provide consistent outcomes, so that both the data itself and the results contribute to a valid overall interpretation. Due to the relatively low number of events, we were unable to develop a model to estimate a corridor of TAC levels that would not increase the risk of CLAD10.

## Conclusion

In summary, our results suggest that a potentially relevant sequence of events leading to CLAD10 may exist, which could be mitigated by specific interventions aimed at maintaining appropriate immunosuppression during phases of AKI, and mandate the need for sensible rules on how to handle calcineurin-inhibitor-induced kidney injury. Our findings emphasize that even a relatively short-term reduction in TAC-based immunosuppression may be sufficient to trigger the development of CLAD10. The limitations of our study necessitate further prospective clinical trials and validation.

## Data Availability

The raw data supporting the conclusions of this article will be made available by the authors, without undue reservation.
