# Supplementary material for: The critical relationship between tacrolimus levels, acute kidney injury, and early chronic lung allograft dysfunction
Source: Front Transplant. 2026 Jan 7;4:1704682. doi: 10.3389/frtra.2025.1704682 (PMC12819767; doi:10.3389/frtra.2025.1704682)
Supplement: Supplementary file 1 [file Supplementaryfile2.pdf]

Supplement: The critical relationship between tacrolimus levels, acute kidney injury, and early chronic lung allograft dysfunction

Roman Hauber[1*], Luca Kohlhepp[2*], Ignaz Briegel[3*], Tobias Veit[3], Jürgen Barton[3], Bruno Meiser[4], Christian Schneider[5], Teresa Kauke[5], Rudolf Hatz[5], Dominik J. Hoechter[6], Nikolaus Kneidinger[7], Jürgen Behr[3]

1. Department of Internal Medicine II, Neuwittelsbach Academic Hospital of the University Hospital LMU Munich, Munich, Germany.
2. Center for Artificial Intelligence and Data Science, JMU University of Würzburg, Würzburg, Germany
3. Department of Medicine V, Comprehensive Pneumology Center Munich (CPC-M), German Center for Lung Research (DZL), University Hospital LMU Munich, Munich, Germany
4. Transplant Center, University Hospital LMU Munich, Munich, Germany
5. Division of Thoracic Surgery, University Hospital LMU Munich, Munich, Germany
6. Department of Anaesthesiology, University Hospital LMU Munich, Munich, Germany
7. Division of Pulmonology, Department of Internal Medicine, Lung Research Cluster, Medical University of Graz, Graz, Austria

*Authors contributed equally

Introduction

The analysis of patients who died within the first two years after LTx showed that findings were particularly strongly contrasted in this subgroup.

Some patients have multiple laboratory measurements prior to the diagnosis of CLAD10 and some patients have not been measured shortly before CLAD10. This might be used as an additional surrogate marker to distinguish between two groups of patients. Patients who were measured more frequently before the onset of CLAD10, e.g. because they were hospitalized or showed symptoms, may show a different risk profile compared to the overall patient population. Thus, two profiles can be seen: Patients having several measures shortly before CLAD10 (high risk profile) and all patients (overall risk profile). On the one hand this distinction allows the detection of vulnerable patients and on the other hand this approach leads to a potential larger amount of patient data.

In this supplement the question is pursued whether the interaction between tacrolimus (TAC), glomerular filtration rate (GFR) and the onset of CLAD10 can be confirmed using extended risk stratification.

Methods

The analysis for vulnerability is based on the 346 patients who developed CLAD10. The high risk profile is defined as the group of patients who several measurements (≥2) shortly before the onset of CLAD10. The overall risk profile includes all other patients, including those who had no data shortly before CLAD10.

We used data from a 90 days interval prior to CLAD10. In Germany, 90 days is a billing-relevant period. Within this period most patients are expected to visit their GP or an outpatient clinic for blood sampling if necessary. Clinically relevant abnormalities or measurement deviations should be visible and checked by short-interval follow-up measurements if necessary. The analysis was also carried out for the 60-day time interval as in the paper to visualize the effect over time.

For our analysis we used univariate and multivariate linear regression models with the standardized FEV1 and time to CLAD10 (TtC) as dependent variables, GFR and TAC as independent variables. To keep the measure of FEV1 more robust, we standardized the FEV1 measured in the period under consideration to the individual best FEV1 - (ind. FEV1/ ind. best FEV1= standardized FEV1).

Results

Figure S1 provides a comprehensive summary of the regression results.

Univariate regression for GFR on standardized FEV1: Analysis for influence of GFR on standardized FEV1 showed the GFR to be significantly associated with the standardized FEV1.

In the high risk profile: A low GFR tends to be associated with a higher FEV1 - $\beta_{GFR60,90}$= -0.1219 (data of 48 patients, 80 data points, $p<0.05$), -0.0746 (data of 124 patients, 209 data points, $p<0.01$)

Univariate regression for TAC on standardized FEV1: TAC shows a significant association with the adjusted FEV1.

In the high risk profile: A higher TAC level tends to be associated with a higher FEV1 – β_{TAC90}= 0.2833 (data of 124 patients, 209 data points, $p<0.05$)

In the overall risk profile: Higher Tac tends to be associated with a higher FEV1 – β_{TAC90}= 0.3256 (data of 299 patients, 481 data points, $p<0.01$)

Multivariate regression for GFR and TAC on FEV1: A multivariate regression for TAC and GFR on FEV1 showed significant coefficients 90 days prior to CLAD10.

In the high risk profile: A higher TAC level and a lower GFR are associated with a higher FEV1 in the combined model - β_{TAC90}= 0.2728, β_{GFR90}= -0.0731 (data of 124 patients, 209 data points, $p<0.05$)

Univariate regression for TAC on the time to CLAD10 (TtC): Both GFR and TAC showed significant effect estimates in the linear regression model with regard to time to CLAD10.

In the high risk profile: Lower TAC tends to be associated with a longer time to CLAD10 – $\beta_{TAC60,90}$= 0.6173 (data of 75 patients, 119 data points, $p<0.05$), 0.9613 (data of 192 patients, 333 data points, $p<0.01$)

In the overall risk profile: A high GFR tends to be associated with a longer time-to- CLAD10 – $\beta_{GFR60,90}$= -0.1262 (data of 290 patients, 391 data points, $p<0.01$), -0.1326 (data of 299 patients, 481 data points, $p<0.05$). Higher Tac tends to be associated with a longer time-to- CLAD10 – $\beta_{TAC60,90}$= -0.7911 (data of 290 patients, 391 data points, $p<0.001$), -1.1548 (data of 299 patients, 481 data points, $p<0.001$).

Discussion

Applying basic linear regression models while focusing on different risk profiles allowed to analyze more patients who are likely to have a comparable risk profile. The overall findings of high-risk patients in this supplement are in line with the findings in the paper, namely the existence of a potentially relevant sequence of events before the onset of CLAD10. Yet the results are partly counterintuitive. Possible explanations for these results are described below, but due to the lack of clarity, we refrain from taking this interpretation as a given and do not draw a strong conclusion from the results shown.

Standardized FEV1

In the results a low GFR is significantly associated with a higher FEV1. At the same time, a higher TAC level shows to be associated with a higher FEV1. No significant effect between TAC and GFR could be found. The multivariate regression shows that increased TAC and decreased GFR are jointly associated with increased FEV1 in the multivariate model. In the light of these findings, it may hypothesized that a higher TAC level lowered the GFR as known in the literature but cannot be ascertained. We see an association between bad kidney function and good lung function, which seems counterintuitive. We consider the correlation between low GFR and high FEV1 to be biased and potentially influenced by the TAC level, acting as a mediator variable. The fact that no correlation can be seen between TAC and GFR may be due to short-term fluctuations in the TAC level and a time shift, which is not visible in the regression.

Time to CLAD10

FEV1 is seen as a surrogate marker for the occurrence of CLAD10. In our study, GFR shows a negative association with FEV1 and at the same time GFR shows a positive association with time to CLAD10. Due to the contradictory trends this result highlights that the FEV1 has limited use for the assessment of the time to CLAD10.

High risk profile and the overall risk profile show a different association between TAC and time to CLAD10. While in high-risk patients a higher TAC is associated with a shorter time to CLAD, in the overall risk profile a higher TAC level is associated with a longer time to CLAD10. This finding matches the results of our study that some effects only contrast in specific groups. The association of the high-risk

group is consistent with the analysis of patients who died within the first two years. We hypothesize that these patients may have had particular disease burden with renal imbalances or possibly inadvertently elevated TAC levels, which may be associated with a more rapid onset of CLAD10. However, a causal attribution and a strong conclusion are not possible.

Conclusion

The supplementary analysis with risk profile definitions provides insights into the complex interactions between lung, renal, and immunosuppression. The screening failures show expected and explainable differences. The results suggest consistency with the main results, but with limitations, so strong conclusions are not drawn.

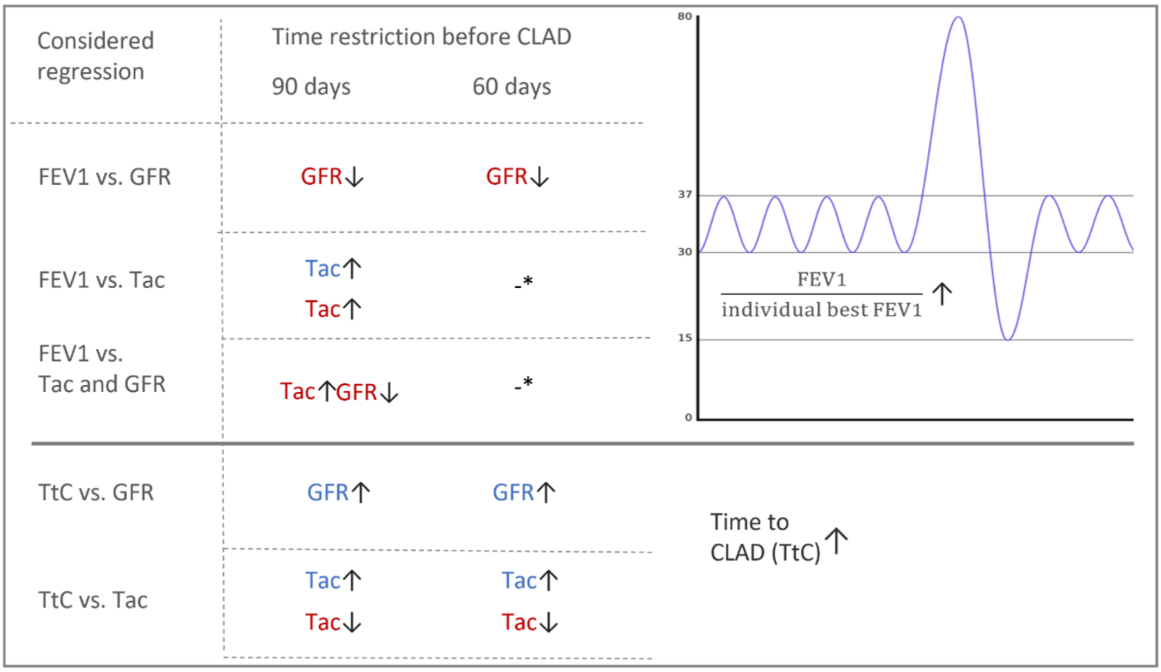

Figure S1 Visualization of the regression correlations in the period of 60 and 90 days before CLAD10. The influencing variables considered are GFR and TAC level. Dependent variables are standardized FEV1 and time-to-CLAD10 (TtC). The sense of the correlation and the risk profile is shown. High risk profile: red, Overall risk profile: blue. Non-significant correlation: -.*
